# Characterization of Fine Metal Particles Derived from Shredded WEEE Using a Hyperspectral Image System: Preliminary Results

**DOI:** 10.3390/s17051117

**Published:** 2017-05-13

**Authors:** Gabriele Candiani, Nicoletta Picone, Loredana Pompilio, Monica Pepe, Marcello Colledani

**Affiliations:** 1 Institute for the Electromagnetic Sensing of Environment, National Research Council, 20133 Milano, Italy; pepe.m@irea.cnr.it; 2 Institute of Industrial Technologies and Automation, National Research Council, 20133 Milano, Italy; nicoletta.picone@itia.cnr.it (N.P.); marcello.colledani@polimi.it (M.C.); 3 Department of Psychological, Health and Earth Sciences, G. D’Annunzio University, 66031 Chieti, Italy; loredana.pompilio@unich.it; 4 Department of Mechanical Engineering, Polytechnic of Milan, 20156 Milano, Italy

**Keywords:** hyperspectral sensor, fine metal particles, WEEE recycling

## Abstract

Waste of electric and electronic equipment (WEEE) is the fastest-growing waste stream in Europe. The large amount of electric and electronic products introduced every year in the market makes WEEE disposal a relevant problem. On the other hand, the high abundance of key metals included in WEEE has increased the industrial interest in WEEE recycling. However, the high variability of materials used to produce electric and electronic equipment makes key metals’ recovery a complex task: the separation process requires flexible systems, which are not currently implemented in recycling plants. In this context, hyperspectral sensors and imaging systems represent a suitable technology to improve WEEE recycling rates and the quality of the output products. This work introduces the preliminary tests using a hyperspectral system, integrated in an automatic WEEE recycling pilot plant, for the characterization of mixtures of fine particles derived from WEEE shredding. Several combinations of classification algorithms and techniques for signal enhancement of reflectance spectra were implemented and compared. The methodology introduced in this study has shown characterization accuracies greater than 95%.

## 1. Introduction

According to recent studies, waste of electrical and electronic equipment (WEEE) is considered the fastest-growing waste stream in the European Union (EU), with an increasing rate of 3–5% per year, which is expected to grow to more than 12 tons by 2020 [1]. Due to the large amount of new electrical and electronic equipment introduced every year in the market and the wide variety of materials used to manufacture these products, including hazardous content, WEEE can represent an environmental and health problem when not correctly managed. Unfortunately, the high variability of WEEE composition makes the recycling process a very complex task. According to the latest Eurostat review [2], only one third of WEEE is recycled and reused. The remaining fraction is either collected by unregistered enterprises or disposed in landfills or incinerators as part of residual waste. Nevertheless, electrical and electronic equipment is often made of rare and expensive key metals. For example, printed circuit boards (PCBs) are an important fraction of the so-called urban mining resources, since they are composed of 25–30% (by weight) of valuable metals such as copper, iron, brass, tin, nickel, gold and silver. Therefore, proper collection and recycling can become a very important source of key metals for advanced technological products, improving the urban mineral exploitation and enhancing the circular economy. From these considerations, it is clear how WEEE constitutes both a supply problem and a market opportunity.

In order to deal with the main topics related to WEEE—fostering an advantageous exploitation, mitigating the cost growth of raw materials, as well as reducing supply shortage risks for the key metals used in high-technology applications—many countries have introduced laws aimed to improve material recycling rates. The first European Recycling Directive on WEEE (Directive 2002/96/EC; [3]) has been in force since February 2003. The legislation provides for the creation of collection schemes where consumers return their used waste equipment free of charge. In particular, the directive states that all EU countries have to recover about 70–80% by weight of the produced WEEE and to reuse 50–75% of the recovered materials or components. In order to tackle the fast increase of the WEEE stream, a revised WEEE Directive (Directive 2012/19/EU; [4]) entered into force in August 2012, and it is effective from February 2014.

Since the introduction of the WEEE Directive, the interest of companies in WEEE treatment has dramatically increased. In the European recycling industry, 85% of these companies are small and medium enterprises (SMEs), which despite the high variability and the continuous evolution of electrical and electronic products, do not evolve the employed mechanical processes, accordingly. The lack of adaptive systems, coupled with the high variability in input materials, ultimately has caused: (i) poor recycling rates, especially for key metals; (ii) the overuse of landfills also for materials that are potentially recyclable; and (iii) the lack of competitiveness of SMEs due to the low purity of recycled materials.

In this context, the integration of hyperspectral imaging (HSI) systems into WEEE recycling plants constitutes a suitable solution to help overcome the lack of adaptability of the processes in use. An in-line mixture characterization, performed through the HSI technology, can enable a flexible management of the WEEE recycling process. The knowledge of either the percentage of different key metals or the ratio metals-to-non-metals inside the shredded mixture is a relevant piece of information at many stages in the process: (i) before the sorting step, it can drive the selection of the best set of parameters for the separation process; (ii) after the sorting step, it allows measuring the performance of the separation phase; and (iii) at the end of the whole recycling process, it can ground the assessment of output quality.

Despite the aforementioned advantages related to the integration of hyperspectral vision systems into WEEE recycling plants, the scientific literature presents very few examples of their practical/experimental use. The majority of contributions is related to the cooperative research project SORMEN [5], a project devoted to the development of a new technology based on multispectral and hyperspectral characterization of WEEE mixtures for the separation of non-ferrous metal particles. In particular, [6,7,8,9] describe the main outcome of the project: a framework for the automatic sorting of non-ferrous materials exploiting hyperspectral data. The authors report the results obtained testing a new technique for the decorrelation of hyperspectral data and a classification algorithm, which integrates the spectral and spatial features of particles. Even if the results achieved in these works show classification rates up to 98%, the developed solution enables characterizing WEEE coarse particles with sizes ranging from 10–50 mm. The main constraint to an actual industrial exploitation is that usually, the recovery of high value materials (key metals and rare earth) requires very fine shredding in order to obtain single-material particles.

Previously, Kutila et al. [10] introduced a novel automatic sorting system for scrap metals based on a color vision system and an inductive sensor array. The test conducted in a real metal recycling plant proved that an 80% separation purity level can be achieved only when reddish (copper, brass) and white metals (stainless steel) are separated.

More recently, Picone et al. [11] proposed the use of a near-infrared (NIR) hyperspectral camera to sort the very fine particles (lower than 2 mm) of copper and PVC, derived from shredded electrical wires. The paper showed classification rates greater than 94% and 95% for copper and plastics, respectively, using a classification model based on partial least square-discriminant analysis (PLS-DA).

Barnabè et al. [12] introduced a prototype system, combining two hyperspectral cameras, one operating in the visible and near-infrared (VNIR) spectral range and the other one in the short-wavelength infrared (SWIR), for the characterization of metallic fine-sized particles, coming from end-of-life vehicles and WEEE. The system was used to classify a dataset of 100 metallic fragments, previously characterized with XRF technology.

In Candiani et al. [13], the authors tested the performance of an HSI technology for WEEE sorting, comparing several combinations of (i) classification algorithms and (ii) methods for the compensation of illumination conditions. The latter is a very crucial point since such conditions highly affect the acquired signal of metal particles, as explained in the following sections. The authors characterized a mixture of very fine particles (lower than 2 mm) of brass, iron and copper, achieving classification accuracies greater than 80% using a hyperspectral camera working in the VNIR spectral range.

This work features an extension of the results obtained in [13], introducing a new dataset, which increases the number of metals to be characterized. In particular, this study evaluates the capability of the VNIR hyperspectral camera to characterize mixtures composed of particles from five different metals: besides brass (CuZn), iron (Fe) and copper (Cu), particles of aluminum (Al) and nickel (Ni) were added to the dataset. Several combinations of illumination-compensation methods and classification algorithms were tested to evaluate their performance for metal characterization. A post-classification step was included to exploit spatial information derived from the assumption of homogeneous particles. This step helped to increase the classification accuracy form 80% up to 95%. Moreover, since the VNIR hyperspectral camera is integrated with an automatic WEEE recycling pilot plant, a sensitivity analysis has been carried out on the data compression procedure, a very relevant step when dealing with an in-line system.

The paper is structured as follows:

- Section 2: (i) gives an overview of the whole system, describing the pilot plant for de- and re-manufacturing developed at the Italian National Research Council (CNR) and the vision system setup and integration into the pilot plant; (ii) introduces the dataset used in this study; and (iii) describes the processing steps followed for the characterization of mixtures;
- Section 3 makes apparent the results achieved in this study, discussing the performance of the HSI system and the implemented procedure, highlighting advantages and disadvantages;
- Section 4 resumes the results, draws the conclusions and introduces possible future works.

## 2. Materials and Methods

### 2.1. System Overview

The analysis presented in this study was performed at the de- and re-manufacturing Pilot Plant developed at the Institute of Industrial Technology and Automation (ITIA) of the Italian National Research Council (CNR). The ITIA-CNR Pilot Plant consists of three cells for the end-of-life treatment of mechatronic products (i.e., PCBs): Cell-1, disassembly; Cell-2, reworking; Cell-3, recycling. Whereas the first two cells are dedicated to the semi-automated human-robot cooperation solutions for PCBs’ disassembly and reworking, the third cell is devoted to the recycling of products and components that cannot be reconditioned. In Cell-3, PCBs are mechanically treated through coarse and fine shredding processes in order to obtain a mixture of particles. This mixture is then separated either with (i) a corona electrostatic separator (CES), for conductive/non-conductive fractions, or through (ii) an eddy current separator (ECS), in the case of ferrous/non-ferrous and non-metallic fractions. Cell-3 includes also the vision system used for in-line characterization of the mixture derived from shredded PCBs. The feedback provided by the HSI system in terms of metal and non-metal fractions allows the optimization of working parameters for the separation processes and the triggering of control models for both CES and ECS (i.e., electrode voltage, drum speed, material feed rate and output stream splitter position), according to the specific type of mixture under treatment. Lastly, all components in Cell-3 are linked through a pneumatic transport system, which ensures the run-time reconfiguration of particles’ routing, enabling the flexibility needed in WEEE recycling processes.

### 2.2. Vision System Features and Setup

**Figure 1:**
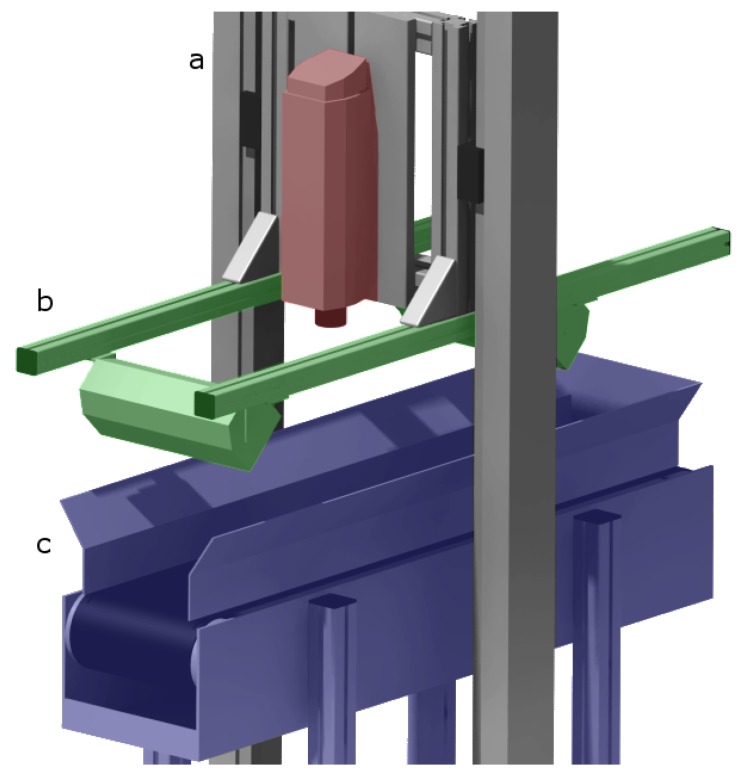
Rendering of the vision system: (**a**) hyperspectral camera (red); (**b**) illumination system (green); (**c**) transport system (blue).

The vision system integrated in the ITIA-CNR Pilot Plant (Figure 1) includes three main elements: (i) the hyperspectral camera (Figure 1a); (ii) the illumination system (Figure 1b); and (iii) the transport system (Figure 1c). In particular, two hyperspectral cameras are available at the Pilot Plant: both can be individually mounted on the support and swapped according to the type of materials to be analyzed. The VNIR camera used in this study is the PFD model from Specim. It consists of an ImSpector™ V10E, covering the spectral range range 400–1000 nm, and a high speed CMOS detector. The CMOS sensor can register 1312 spatial pixels in 768 spectral bands at a frame rate of 65 fps. The camera features a spectral binning up to 8× (98 spectral bands). This feature allows adjusting the spectral sampling from 0.78 nm/pixel–6.27 nm/pixel and to increase the frame rate up to 100 fps. The camera is equipped with an OLE23 fore objective lens with a focal length of 23 mm and an FOV of $25.7^{\circ }$. The NIR camera is the VLNIR model from Specim. It integrates an ImSpector™ N17E imaging spectrograph working in the range 900–1700 nm and a temperature-stabilized TE-cooled InGaAs photodiode array. The camera features a spatial resolution of 320 pixels and a spectral resolution of 240 bands, with a spectral sampling of 3.3 nm. The images are recorded at 12 bits with a fixed frame rate of 100 fps. The camera is equipped with a 31-mm lens and a field of view of 130 mm. Both cameras are controlled by a PC unit equipped with the SpectralDAQ software [14]: this software allows one to setup the camera parameters, acquire the data and visualize the image in real time. The illumination is provided by two opposite slots housing 3 halogen lamps each, which emit in the spectral range 380–2200 nm. The transport system consists of a moving conveyor belt, made of black PVC. The conveyor measures 20 cm in width and 100 cm in length, and its speed can be externally adjusted from 0–4 m/min. A vibrating feeder is responsible for the deposition of waste material on the conveyor belt, in such a way to avoid particles’ overlap. The feeder frequency can be adjusted according to the particles dimension, within a range from 0–50 Hz.

The setup of the vision system was optimized in order to support the in-line characterization of waste materials in an actual recycling plant, balancing the speed of the conveyor, the imaging frame rate and binning to allow the timely processing of acquired hyperspectral images for the recognition of fine metal particles. According to these requirements, the VNIR camera was mounted at approximately 30 cm above the transport system: at this distance, the FOV provides a swath of 13 cm with a pixel pitch of 0.1 mm, which is the maximum spatial resolution achievable by the camera. The spectral binning value was set to 8×, enabling the acquisition of images in 98 spectral bands at the maximum frame rate of 100 fps. The lamps were mounted with a tilt angle of $45^{\circ }$, in a configuration known as “linear dark field lightning”. This configuration features a low to medium angle of incidence and typically requires close proximity. Dark field configuration relies on the fact that most of the light incident on a shiny surface is reflected away from the camera, minimizing effects such as hot spot glares. The relatively low amount of light reflected back into the camera is due to the roughness, imperfections or small features of the surface [15]. Nonetheless, due to the complex nature of shredded metal particles, characterized by irregular three-dimensional shapes with both rough and high reflective surfaces, the acquired images were affected by heterogeneous illumination and shading effects including shadows and specular reflections. These effects need to be compensated in order to minimize the possible sources of error in the classification process (see Section 2.4).

### 2.3. Experimental Data

**Figure 2:**
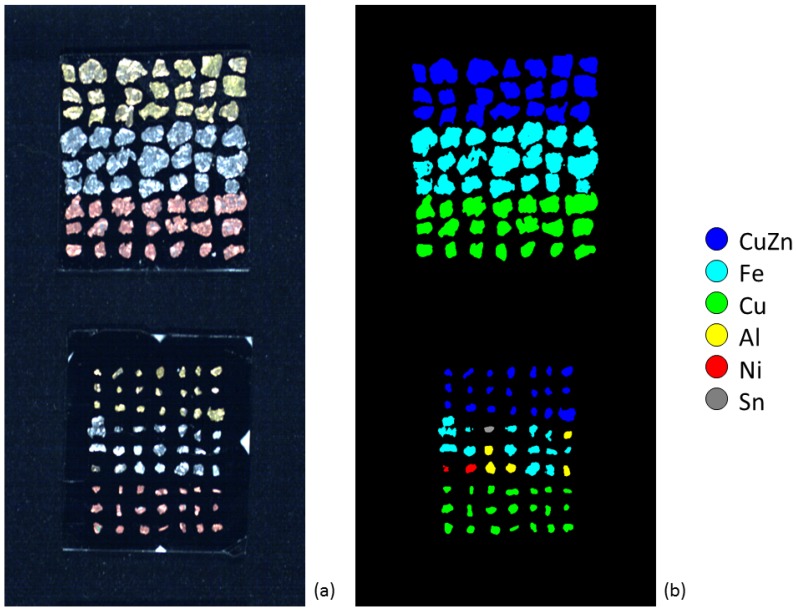
The dataset used in this study: (**a**) true colors image of the metal particles acquired through the VIS hyperspectral camera; (**b**) color-coded image in which each particle is associated with a single metal according to their average chemical composition measured through a scanning electron microscope (top: sample ID002; bottom: sample ID001).

In this study, several fine metal particles (average size smaller than 2 mm) were hand-picked from WEEE shredded PCBs, sorted according to their color and size, and then attached on two different 5 cm ×5 cm tiles, using patches of bi-adhesive black tape. The particles were arranged on both tiles in a grid configuration of 9×7: the particles with a size lower than 1 mm were attached to the first tile (Figure 2, sample ID001), whereas the second tile collected the particles with sizes ranging from 1 mm–2 mm (Figure 2, sample ID002).

Previous laboratory measurements were carried out on foils made of pure metals (above 99.5% purity) using a Spectral Evolution RS-3500 spectroradiometer (spectral range covering 350–2500 nm; spectral resolution ranging from 3–8 nm) equipped with a contact probe. These measurements revealed that the investigated metals are almost featureless within the whole 350–2500-nm wavelength interval, except for some weak features in the 400–1000-nm range. For this reason, the VNIR camera was chosen to acquire hyperspectral images of the tiles containing metal particles, dark current $B\left(\lambda \right)$ and white reference $W\left(\lambda \right)$, using the configuration described in the previous section. The dark current image was recorded closing the camera shutter (i.e., measuring the camera noise), whereas the white reference was obtained acquiring an image of a white tile 300 mm × 25 mm × 10 mm in size, made of Spectralon^®^. This is a fluoropolymer used for the construction of optical components, such as calibration targets, which features a highly Lambertian behavior and the highest diffuse reflectance of any known material or coating, along the ultraviolet, visible and near-infrared range.

The particles of both samples (ID001 and ID002) were also analyzed with a scanning electron microscope (SEM). The SEM uses a focused beam of high-energy electrons to retrieve several types of information. The interaction between the electrons generated by the microscope and the atoms of the sample produces various types of signals, which are related to the sample external morphology (texture) and chemical composition. Detectable signals include secondary electrons (SE), back-scattered electrons (BSE), characteristic X-rays, cathodoluminescence (CL), current and transmitted electrons. In this study, the chemistry of each particle was measured from the BSE signal emerging from a wide area of the particle surface. Since the energy of the BSE is strongly related to the atomic number of a specimen, the SEM analysis provided the composition and abundance of chemical elements in each particle. The results of SEM analyses (Table 1) show that each metal particle, obtained after the shredding process, was not composed of pure metal. The presence of other materials can range from 10%–23% in the class composition, with a degree of uncertainty. Nevertheless, each particle was assumed to be homogeneous and associated with its most abundant metal class (Figure 2). Even if this assumption certainly has an impact on the mixture characterization, it can be justified by considering these minor materials as impurities, because of their low abundance. This contamination can be due to several effects, such as: (i) weathering (i.e., metal oxidation); (ii) mechanical processing (i.e., friction against other materials); (iii) a flawed separation of single metals during the shredding process; (iv) uncertainties of the SEM measurements; and/or (v) the use of additives in order to improve the mechanical and chemical properties of metals or alloys. For instance, small amounts of Al are added to make brass harder and corrosion resistant, whereas Sn is used as well for the same reasons, especially for seawater applications. Nevertheless, in order to correctly attribute the training samples in the spectral classification process, it was necessary to identify each particle with the most abundant metallic specimen in its bulk composition. Considering the average composition of PCBs, five metals/alloys among those with the highest concentrations were selected in this study: CuZn, Fe, Cu, Al and Ni. Although identified, Sn was not used because the only found particle was too small to provide a significant amount of pixels for the selection of training and test samples.

### 2.4. Data Processing

The in-line WEEE particle characterizations were performed by combining several classification algorithms and different methods for the compensation of non-uniform illumination: overall, a total of 16 different classification/compensation configurations were tested and compared. The whole processing chain adopted in this study included the following steps:

1. image calibration;
2. data compression;
3. illumination compensation;
4. training and test samples selection;
5. pixel-wise classification;
6. particle-wise classification;
7. accuracy assessment.

In the first step, the raw image DN(λ,x) acquired with the VNIR hyperspectral camera was calibrated for the variable intensity of incident light caused by the spatial heterogeneity of the illumination system in the across-track direction *x*. This step was performed using the previously-measured images of dark current B(λ,x) and white reference W(λ,x), through the following equation:

(1) $$R\left(\lambda \right)=\frac{DN\left(\lambda ,x\right)-B\left(\lambda ,x\right)}{W\left(\lambda ,x\right)-B\left(\lambda ,x\right)}$$

where λ represents the wavelength of spectral bands. The resulting reflectance $R\left(\lambda \right)$ is thus calibrated for illumination heterogeneity, loosing the dependence from spatial direction *x*. Moreover, in order to avoid data with a poor signal-to-noise ratio, the first and last bands of the sensor were removed, resizing the original spectral range to 500–900 nm.

**Figure 3:**
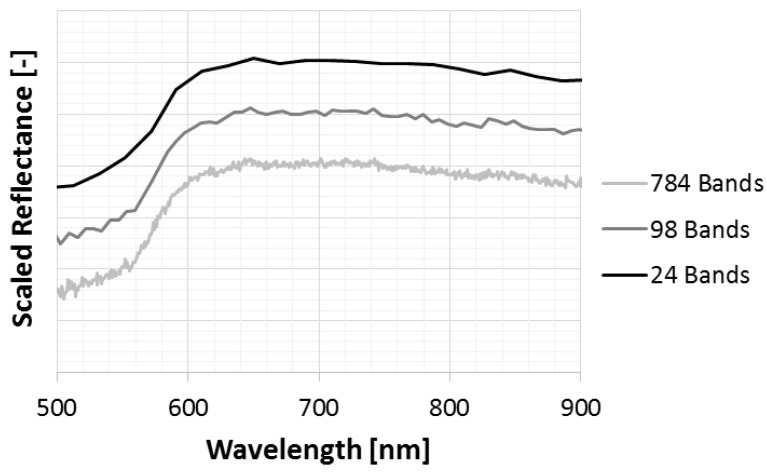
Example of the 8× binning internally performed by the camera software SpectralDAQ (from 768 down to 98 bands) and data compression performed with the triangular fuzzy sets (from 98 down to 24 bands) over a Cu spectrum (reflectances scaled for clarity).

Since the high number of bands provided by hyperspectral sensors leads to a high level of redundancy and correlation, several previous works proposed data reduction methods based on principal component analysis (PCA) [16], linear discriminant analysis (LDA) [17], wavelet transformation [18], automatic band selection [19] and spectral fuzzy sets [6]. In particular, in [6], the spectral fuzzy sets method was compared with other feature reduction techniques (based on PCA, LDA and wavelet decomposition, among others). The comparison has shown that fuzzy sets outperformed the other methods, improving both computational efficiency and classification accuracy. According to this method, the membership value of each band is defined through a triangular function centered at the wavelengths of the new reduced bands. The membership value is maximum at these wavelength positions and linearly decreases moving farther: this means that bands closer to the new wavelengths have greater weights than farther ones. Moreover, this method is useful to reduce spectral noise: the triangular membership functions act like a filter, smoothing the reflectance values of the reduced bands (Figure 3). For these reasons, this method has been employed in this study to reduce the original 98 bands to a new set of 24 bands. Since the number of fuzzy sets can be critical, a sensitivity analysis was carried out (see Section 3.2): several characterization tests were performed varying the number of fuzzy sets from 4–24. The goal of this analysis was to understand how the number of fuzzy sets affects the mixture characterization and to find an optimal trade-off between computing performance and classification accuracy.

**Figure 4:**
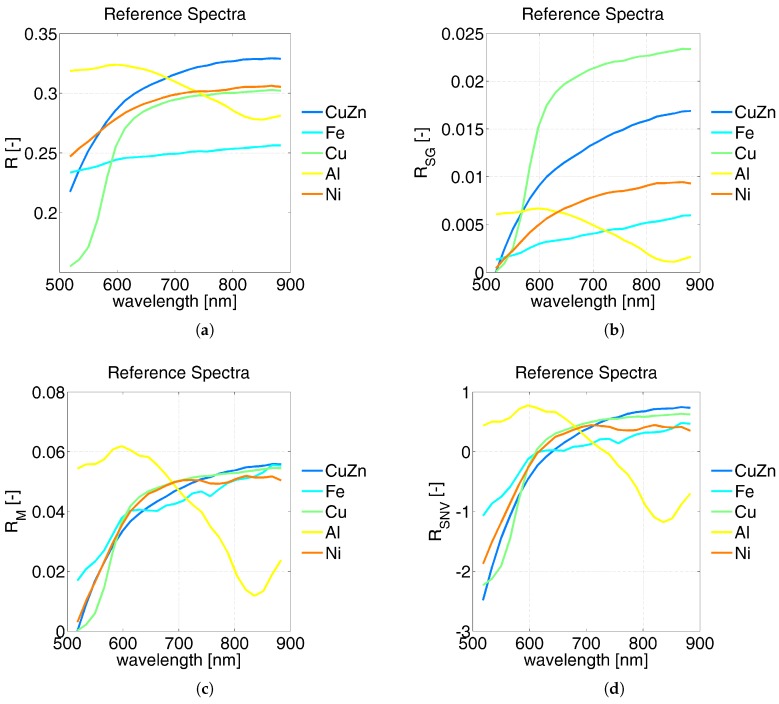
Example of the effects of different techniques for illumination compensation. Spectra represent the average reflectances of the training samples selected for this study (average of 100 random pixels for each metal). (**a**) No compensation (*R*); (**b**) Stokman and Gevers ($R_{SG}$); (**c**) Montoliu ($R_{M}$); (**d**) standard normal variate (SNV) ($R_{SNV}$).

As observed in [8], beside heterogeneity due to the illumination system, the image acquisition process of metal particles in a laboratory is highly affected by shading effects, such as shadows and specular reflections (highlights). To compensate for these effects, two methods originally proposed in Stockman and Gevers [20] and Montoliu [21] were tested, along with the standard normal variate (SNV) algorithm:

(2) $$R_{SG}\left(\lambda \right)=\frac{R\left(\lambda \right)}{\sum_{i=1}^{N}R\left(\lambda_{i}\right)}-\min_{j\in \left[1,N\right]}\frac{R\left(\lambda_{j}\right)}{\sum_{i=1}^{N}R\left(\lambda_{i}\right)}$$

(3) $$R_{M}\left(\lambda \right)=\frac{R\left(\lambda \right)-\min_{j\in \left[1,N\right]}R\left(\lambda_{j}\right)}{\sum_{i=1}^{N}\left(R\left(\lambda_{i}\right)-\min_{j\in \left[1,N\right]}R\left(\lambda_{j}\right)\right)}$$

(4) $$R_{SNV}\left(\lambda \right)=\frac{R\left(\lambda \right)-\mu_{R}}{\sigma_{R}}$$

where $R_{SG}$, $R_{M}$ and $R_{SNV}$ are the reflectance spectra computed with the methods proposed by Stokman and Gevers, Monotoliu and the SNV, respectively; *N* represents the number of spectral bands; $\mu_{R}$ and $\sigma_{R}$ are the average and standard deviation of the reflectance spectrum. Figure 4 shows how these compensation methods work: the plots illustrate the compensation effects on the average reflectance spectra of each class. These class averages were calculated from the reflectance spectra of training samples, selected as explained in the following step.

Since the classification algorithms tested in this study belong to the supervised category, they require a set of training samples. These samples are used in the learning phase to calculate the statistics of each *k*-th class, such as the average reflectance spectrum (the class reference $r_{k}$), variances and the covariance matrix $C_{k}$. The covariance matrix describes the relationships between reflectance values at different wavelengths for the *K* classes. The selection of training samples was performed exploiting the results of SEM analysis described in the previous section. For each of the 5 metal classes used in this study (Al, CuZn, Cu, Fe and Ni), 100 pixels were selected from the labeled particles, adopting a stratified random sampling strategy: these 100 pixels were used as a reference to train the classification algorithms.

The classification step was performed testing 4 different classification algorithms, for all of the compensation methods previously presented. The classification algorithms were selected among some of the most popular classifiers in the remote sensing domain: the spectral angle mapper (SAM, [22]), the minimum distance (MD, [23]), the Mahalanobis distance (MhlD, [23]) and the maximum likelihood (ML, [23]). These algorithms use different metrics (angular distance, Euclidean distance, probability) to measure the similarity of two spectra (a target and a reference): each target spectrum is associated with the class with the closest reference spectrum, according to the chosen metric. In particular, during the classification procedure, the distances of each pixel spectrum t (target) from all of the *K* reference spectra $r_{k}$ are computed: a pixel is then classified into the *k*-th class whose distance is the smallest (the largest in the case of ML, where the metric is the probability). The equations of the metrics used in these algorithms are briefly described in the following paragraphs.

The spectral angle mapper is a physically-based algorithm, which provides a measure of spectral similarity by calculating the angle between two spectra. Considering a space with a dimensionality equal to the number of bands, the angle θ between a pixel spectrum t and the reference spectrum of the *k*-th class $r_{k}$ can be calculated as:

(5) $$\theta_{SAM}\left(t,r_{k}\right)=\cos^{-1}\left(\frac{\langle t,r_{k}\rangle }{∥t∥\cdot ∥r_{k}∥}\right)$$

The minimum distance algorithm calculates the Euclidean distance between two spectra, t and $r_{k}$, according to the following equation:

(6) $$d_{MD}^{2}\left(t,r_{k}\right)={\left(t-r_{k}\right)}^{T}\left(t-r_{k}\right)$$

The Mahalanobis distance is a measure of similarity, which takes into account the covariances $C_{k}$, but assuming the covariance matrices equal for all of the *K* classes: $C_{1}=C_{2}=...=C_{k}=C$. In practical applications, C can be calculated as the average of the *K* covariance matrices. The Mahalanobis distance is given by:

(7) $$d_{MhlD}^{2}\left(t,r_{k}\right)={\left(t-r_{k}\right)}^{T}C^{-1}\left(t-r_{k}\right)$$

The maximum likelihood algorithm, based on the Bayes theorem, calculates the probability that a pixel t belongs to the *k*-th class. In order to use this algorithm, a sufficient number of reference pixels for each class is required to compute the covariance matrix $C_{k}$. For every pixel t, the probability is then calculated as:

(8) $$d_{ML}\left(t,r_{k}\right)=-\ln\left|C_{k}\right|-{\left(t-r_{k}\right)}^{T}C_{k}^{-1}\left(t-r_{k}\right)$$

It is useful to note that both the Mahalanobis distance and the minimum distance are degenerations of the maximum likelihood algorithm. In particular, the Mahalanobis distance can be derived from the maximum likelihood when considering equal covariances for all of the *K* classes (i.e., $C_{k}=C$ for all *k*), whereas the minimum distance can be obtained with the additional simplification of a diagonal covariance matrix (i.e., $C_{k}=C=\sigma^{2}I$ for all *k*).

As explained in Section 2.3, fine-shredded particles were considered homogeneous (made of single material) due to their small size and the low concentration of other materials. Under this assumption, the results obtained from the pixel-wise classifications were post-processed in order to improve accuracy. Single particles were identified using the function regionprops implemented in the Image Processing Toolbox™ of MATLAB^®^ [24]. To find connected components (i.e., particles), this function: (i) searches for the next unlabeled pixel *p*; (ii) labels all of the pixels in the connected component containing *p* using a flood-fill algorithm; and (iii) repeats the previous steps until all pixels are associated with a connected component. In this step, it is crucial to place the particles separated from each other on the conveyor belt. The mode of all classes included in each connected component was computed and then associated with the relative particle. In other words, each particle was classified with the class presenting the largest number of pixels.

Finally, the performance of all compensation/classification combinations tested in this study was assessed by comparing their results with the reference image consisting of particles labeled on the base of SEM measurements (Figure 2b). The accuracy assessment was performed through the computation of a confusion matrix and its related statistics: the overall accuracy OA, which represents the probability that a pixel randomly sampled from the classification is correctly classified, and the Cohen’s kappa coefficient KC, which takes into account the probability of the agreement by chance [25]. The OA and KC were calculated as follows:

(9) $$OA=\frac{TP}{N}$$

(10) $$KC=\frac{OA-P_{CA}}{1-P_{CA}}$$

where TP are the correctly-classified pixels, *N* is the total number of test samples and $P_{CA}$ is the probability of chance agreement, which incorporates off-diagonal products of row and column totals for each class of confusion matrix.

## 3. Results and Discussion

### 3.1. Mixture Characterization Results

**Figure 5:**
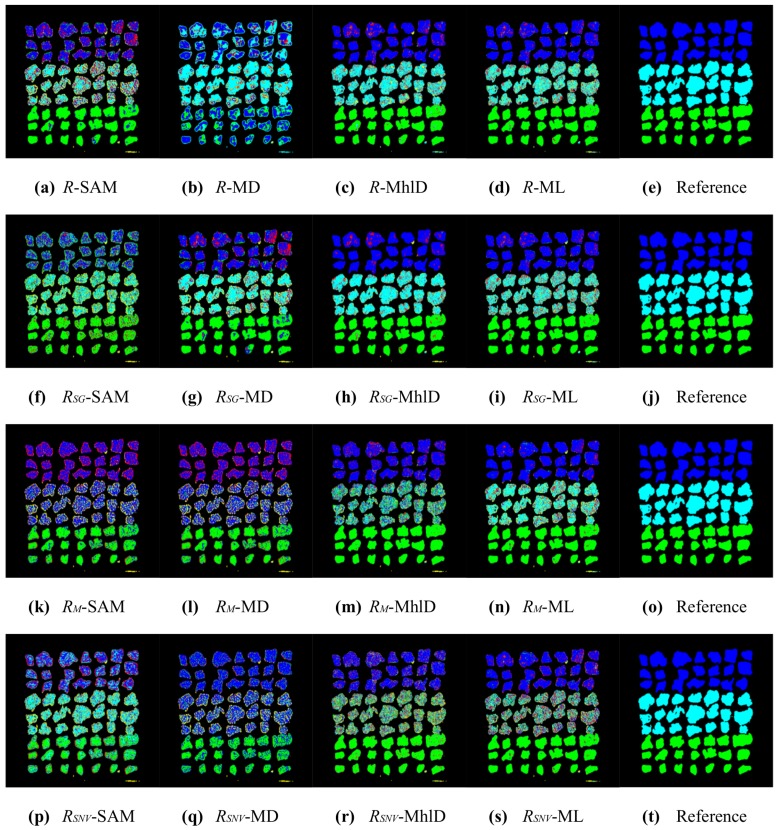
Results of the 16 pixel-wise characterizations for sample ID002 (blue = CuZn, cyan = Fe, green = Cu, yellow = Al, red = Ni, grey = Sn). On the rows (top to bottom): *R* (no compensation), $R_{SG}$, $R_{M}$, $R_{SNV}$. On the columns (left to right): SAM, MD, MhlD, ML; the last column reports the reference image for ease of comparison.

**Figure 6:**
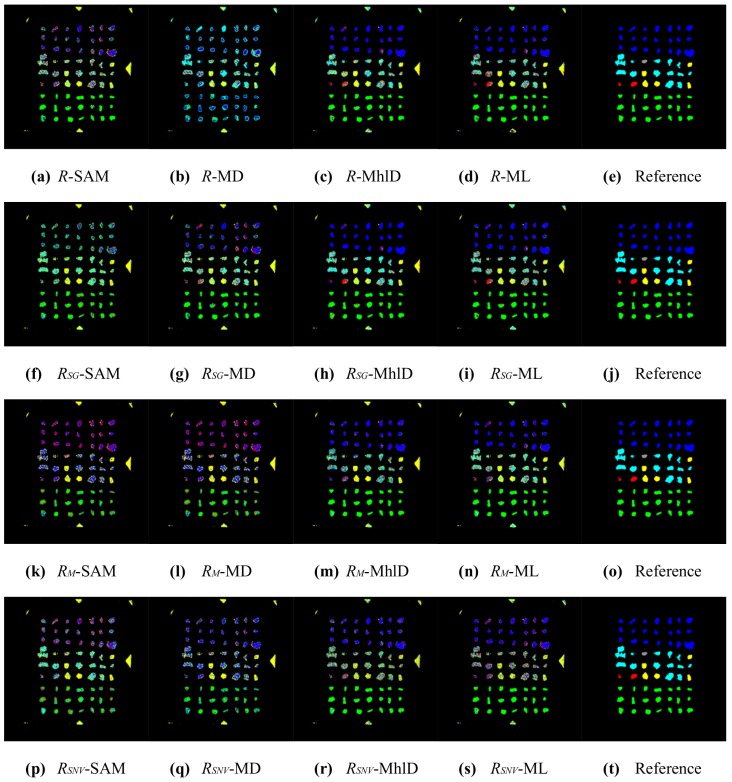
Results of the 16 pixel-wise characterizations for sample ID001 (blue = CuZn, cyan = Fe, green = Cu, yellow = Al, red = Ni, grey = Sn). On the rows (top to bottom): *R* (no compensation), $R_{SG}$, $R_{M}$, $R_{SNV}$. On the columns (left to right): SAM, MD, MhlD, ML; the last column reports the reference image for ease of comparison.

**Figure 7:**
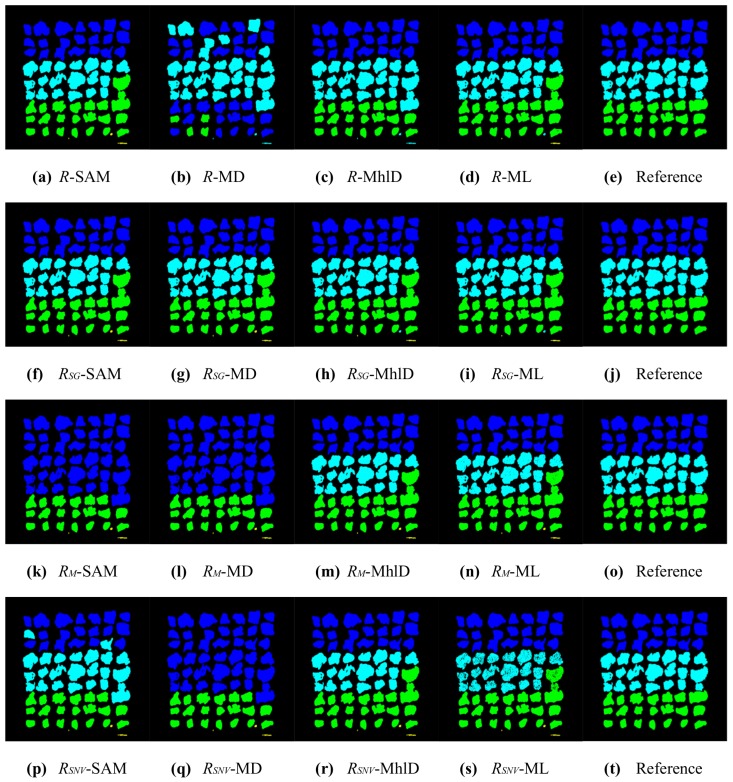
Results of the 16 characterizations for sample ID002, after the particle classification (blue = CuZn, cyan = Fe, green = Cu, yellow = Al, red = Ni, grey = Sn). On the rows (top to bottom): *R* (no compensation), $R_{SG}$, $R_{M}$, $R_{SNV}$. On the columns (left to right): SAM, MD, MhlD, ML; the last column reports the reference image for ease of comparison.

**Figure 8:**
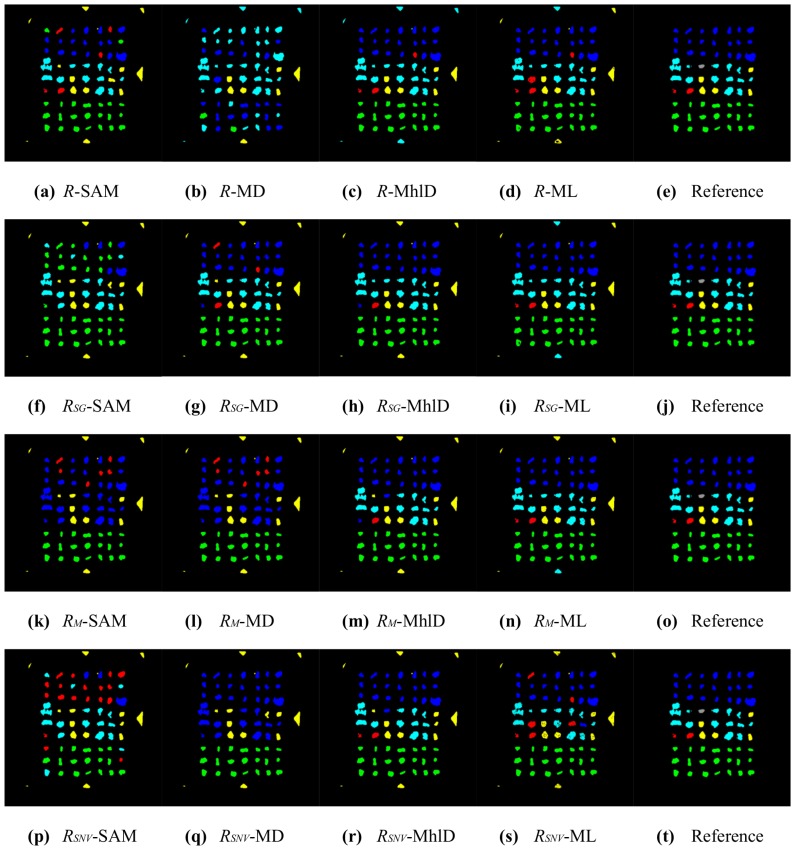
Results of the 16 characterizations for sample ID001, after the particle classification (blue = CuZn, cyan = Fe, green = Cu, yellow = Al, red = Ni, grey = Sn). On the rows (top to bottom): *R* (no compensation), $R_{SG}$, $R_{M}$, $R_{SNV}$. On the columns (left to right): SAM, MD, MhlD, ML; the last column reports the reference image for ease of comparison.

In total, sixteen different mixture characterizations were tested, each resulting from the combination of a classification algorithm (SAM, MD, MhlD, ML) with either raw reflectances *R* or reflectances normalized for illumination effects, using the three compensation methods ($R_{SG}$, $R_{M}$ and $R_{SNV}$) defined in Section 2.4. The classification results are presented in Figure 5, Figure 6, Figure 7 and Figure 8. Table 2 and Table 3 summarize the validation results in terms of overall accuracy OA and kappa coefficient KC.

The validation for the pixel-wise classifications shows that the best results were obtained using algorithms that take into account the spectral relationships through the covariance matrices (MhlD and ML), especially when combined with the $R_{SG}$ compensation or even with raw reflectances *R*, with OA and KC greater than 80% and 0.75, respectively. In general, much lower statistics were achieved with SAM and MD and for both the $R_{M}$ and $R_{SNV}$, due to the many classification errors affecting these combinations.

These results can be partially explained by looking at the plots shown in Figure 4: the method proposed by Stockman and Gevers improved separability among reference metal spectra preserving the spectral shapes. The Montoliu and SNV methods, instead, allowed better distinguishing of the Al spectrum from the others, but they strongly decreased the spectral separability of Fe, Cu, CuZn and Ni. In addition, these algorithms produced relevant changes in the overall spectral shapes of metal particles. Moreover, the illumination effects were not the only source of errors in the classification results. The sensitivity of the classification method to the subtle spectral differences among metals is another important factor, as well as the chemical composition characteristics of the metallic particles tested in this study. The average particle composition, as listed in Table 1, showed a certain variability of metal abundances, due to impurities and contaminations. Therefore, the spectral signatures representative of each class of metal particles spread out over a wide range of reflectances, increasing the spectral variance within each class, especially at NIR wavelengths. Moreover, the spectral analysis of both metal particles and pure metal foils investigated as a reference showed that metals are spectrally featureless, except for Cu and Al, which showed weak broadband absorptions in the VIS and in the NIR range, respectively. As a result, by looking at Table 4, which reports the confusion matrix for the best pixel-wise characterization method ($R_{SG}$-MhlD combination), it can be noticed that Cu and CuZn are the best classified metals along with Al; Fe is sometimes confused with Al and Ni; Al is seldom confused with Fe; Ni scores the worse result, as its spectrum is similar to the spectra of both CuZn and Fe.

The results from pixel-wise classifications were largely improved after the post-processing, where each particle was associated with the modal value of its classes (Figure 7 and Figure 8). The statistics in Table 2 and Table 3 show that classification based on particles increased the accuracy for all 16 combinations. The general trend among the combinations was similar for both pixel-wise and particle classifications: better accuracies were achieved with MhlD and ML algorithms, whereas the $R_{SG}$ compensation method gave the overall best results, even with algorithms such as SAM and MD. In particular, SAM was the algorithm that most took advantage of the particle classification, increasing accuracy from 50–60% to up 90% for three combinations out of four. The best results achieved for pixel-wise classifications, with OA values greater than 80% and KC values greater than 0.75, were improved with particle-wise classification, reaching values greater than 95% and 0.95 for OA and KC, respectively. However, it is worth mentioning that the accuracy of these results strongly depends on the spatial arrangement of particles: in order to get correct results, the particles should not be overlapped and need also to be clearly separate from one another. An example of a classification error caused by close particles can be seen in Figure 7: particles of Rows 5, 6 and 7 in the last column were identified as a single connected object and erroneously classified as the same metal. The confusion matrix for the particle-wise classification with the $R_{SG}$-MhlD combination is shown in Table 5. The main classification error for Fe is related to the proximity of particles, which were badly identified as a single connected object, as mentioned above. The only actual classification error is relative to a Ni particle classified as CuZn.

Considering these results, it can be reasonably stated that methods that take into account the spectral correlation through a covariance matrix, such as the MhlD and MD algorithms, applied to compensated reflectance spectra ($R_{SG}$) are reliable and efficient methods for the classification of metal particles derived from shredded WEEE. The accuracy assessment presented in this paper was in overall agreement with the previous results achieved for the same topic, and presented in [13]. The lower results in pixel-wise classification were somehow expected, since the introduction of new classes tends to increase classification errors, especially when some materials show similar spectral signatures within the considered VNIR spectral range. However, as demonstrated by better accuracy values, the classification at the particle level reduced errors caused by particle impurities, contaminations and errors related to shading effects. Considering the average composition of PCBs (60–65% glass fiber and/or plastic elements, 20% Cu, 8% Fe, 4% Tin, 2% Ni, 2% Pb, 1% Zn, plus other materials with concentrations lower than 0.5%), it is reasonable to expect around 10 classes to be classified for a comprehensive characterization of WEEE mixtures: 6–7 classes for metals and 3–4 classes for other non-metal materials, such as resin and/or plastic materials. In this scenario, it is likely that materials showing very similar reference spectra in the VNIR range will be found, such as some metals and some white-gray plastics. Therefore, in order to improve classification accuracy, it could be worth increasing the spectral range, including the NIR region (900–1700 nm), where resins and plastics show very typical absorption features, and/or even to investigate the UV range, where metals show most of their absorption features.

Another point to take into account is the effect of illumination. The current system, based on two opposite slots containing three halogen lamps each, could not be the best solution for the problem. First of all, it generates a very heterogeneous illumination field, which amplifies the shading effects caused by the three-dimensionality of the particles and by their irregular and rough surfaces. Even if these effects can be partially reduced by image calibration and compensation methods, it could be useful to test a different illumination system. A different option could be represented by diffuse dome lights, which are well suited for curved, specular surfaces or particularly effective at enhancing differentially-angled, textured or topographic features on relatively flat objects. The other possible issue with the current system is the use of halogen lamps. One of the shortcomings of these lamps is the heat flux they produce, which is inherent in the mechanism of light emission itself. However, among the major types of lamps used worldwide, halogen lamps, as well as incandescent and fuel lamps, have emission spectra encompassing the whole VNIR wavelength range, with peak emission in the NIR region. The so-called gas discharge lamps, emitting different series of narrow emission lines, do not fit the requirement of a continuum emission in the wavelength range of interest. The emission spectra of LEDs are highly variable, quite narrow and restricted to visible light [26]. Xenon lamps could be an alternative: they emit a bright white light that is similar to natural sunlight centered in the VIS range, without producing as much heat as the halogen lamps.

### 3.2. Fuzzy Sets Analysis

**Figure 9:**
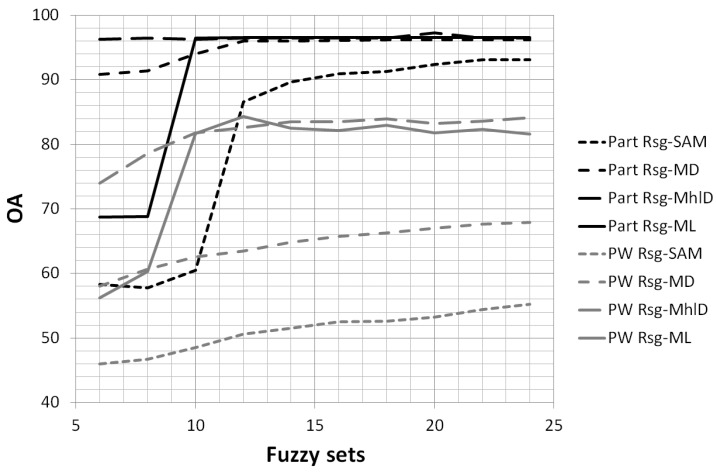
Analysis of the overall accuracy (percent), for pixel-wise (PW) and particle-wise (Part) classifications, achieved changing the number of fuzzy sets from 6–24: examples of the SAM, MD, MhlD and ML algorithms normalized using $R_{SG}$.

As explained in Section 2.4, triangular fuzzy sets were used to compress the amount of data and to reduce data correlation and redundancy. The characterization results previously shown were achieved using 24 sets. However, one might ask if and how the number of fuzzy sets influence the classification results. To answer this question, a sensitivity analysis was performed by changing the number of sets from 6–24. Figure 9 shows the trend of OA for pixel-wise and particle-wise classifications performed using the SAM, MD, MhlD and ML algorithms normalized with $R_{SG}$: the figure shows that accuracies greater than 95% can be achieved starting from 10–12 fuzzy sets for MhlD and ML with both types of classifications. Considering that mixture characterization is integrated in an in-line sorting process, this result is very relevant, demonstrating that high data compression can still guarantee great characterization capabilities and, at the same time, help to speed up processing.

However, it should be taken into account that these results could be valid only for the analyzed metals, which show smooth spectra with subtle absorption features. Further analysis should investigate the sensitivity to the number of fuzzy sets when resin or plastic particles are included in the mixture, in order to understand how the above results can be generalized when applied to materials presenting diagnostic absorption bands.

## 4. Conclusions

Proper treatment of waste from electrical and electronic equipment will become increasingly important in the near future, as it allows reducing the amount of landfill waste and improving the recycling rates of key-metals, thus fostering the circular economy. The current mechanical systems adopted by European SMEs to recycle WEEE are still not flexible enough to manage the high variability of materials contained in electrical and electronic devices. In this framework, the use of vision systems, based on a hyperspectral imaging camera, integrated in WEEE recycling plants, could represent a suitable technology able to improve the flexibility of the recycling processes currently used. Despite its potential benefits, the HSI technology is still not very popular in the WEEE recycling sector. This paper pointed out preliminary results in the characterization of five key metal particles (CuZn, Fe, Cu, Al, Ni) derived from fine-shredded WEEE, using a VNIR hyperspectral camera. The HSI data were pre-processed to obtain reflectance values and then compressed using triangular fuzzy sets. Several combinations of different methods for illumination compensation (Stokman and Gevers $R_{SG}$, Montoliu $R_{M}$ and standard normal variate $R_{SNV}$) and different classification algorithms (spectral angle mapper, minimum distance, Mahalanobis distance and maximum likelihood) were tested and compared. The 16 pixel-wise classifications were post-processed, identifying each particle as a single connected object and then associating it with its most abundant class. The pixel-wise accuracy assessment shows that the best results are achieved with algorithms that take into account spectral relationships, such as MhlD and ML. Among the compensation techniques, only the method proposed by Stokman and Gevers led to classification rates greater than 80%. Quite surprisingly, both the MhlD and ML show high accuracies, even when no illumination compensation was performed. Such classification results highly improved after the post-processing: particle-wise classifications show higher OA and KC values for all 16 combinations, with values up to 97% and 0.96, respectively, for the best combinations. In addition, different configurations of data compression were analyzed changing the numbers of fuzzy sets used to compress the data. The results of this sensitivity analysis show that accuracies greater than 80% can be achieved even for a quite heavy compression rate, increasing the processing speed: the classification rates using only 10–12 fuzzy sets were comparable to the ones obtained with 24 sets.

Even if methods for data compression, illumination compensation and classification could be considered straightforward and the results preliminary, this study demonstrates the usefulness of hyperspectral systems for the classification of particles derived from fine-shredded WEEE. Further studies should be devoted to increasing the number of materials, including those present in PCBs, but not considered in this study, as well as aimed at investigating the potential use of external references to generalize the characterization capabilities of the procedure. Analyses of mixtures containing both metal and resin/plastic particles shall be carried out to further test the validity of the results achieved in this study. Similarly, the sensitivity analysis of the compression rate should be extended to verify its robustness to spectra that show stronger absorption features. Another aspect to investigate is the use of other illumination systems and/or lamps, which can help to reduce heterogeneous illumination and shading effects. Finally, the recycling process could benefit from the use of segmentation or object-oriented techniques, in order to determine the size and shape of the particles in the WEEE mixture, still taking into account the computational costs.

## Figures and Tables

**Table 1:** Average and standard deviation of the chemical composition (weight percentage) for the metal particles measured with SEM.

		Chemical Elements								
		Mg	Al	Si	Fe	Ni	Cu	Zn	Ag	Sn
Particles	Al	1.2%	89.2%	6.5%	1.1%	0.0%	1.2%	0.0%	0.1%	0.8%
		(0.3%)	(3.1%)	(1.1%)	(0.3%)	(0.0%)	(0.9%)	(0.0%)	(0.3%)	(1.1%)
	Cu	3.0%	2.9%	3.6%	0.6%	0.0%	88.0%	0.0%	0.4%	1.2%
		(1.0%)	(2.5%)	(1.5%)	(0.8%)	(0.0%)	(6.9%)	(0.0%)	(1.7%)	(3.6%)
	CuZn	2.4%	2.5%	3.4%	0.8%	2.5%	56.1%	30.9%	0.7%	0.9%
		(0.5%)	(1.1%)	(0.8%)	(0.6%)	(3.8%)	(4.8%)	(1.8%)	(1.4%)	(3.4%)
	Fe	2.1%	2.7%	3.1%	86.2%	3.7%	2.0%	0.0%	0.0%	0.1%
		(0.6%)	(1.7%)	(1.1%)	(6.0%)	(3.9%)	(1.9%)	(0.0%)	(0.0%)	(0.3%)
	Ni	1.5%	4.6%	1.9%	6.6%	76.4%	3.9%	0.0%	0.0%	4.9%
		(0.7%)	(5.4%)	(1.0%)	(9.3%)	(3.5%)	(5.6%)	(0.0%)	(0.0%)	(6.9%)
	Sn	1.3%	1.2%	2.7%	0.0%	4.0%	1.9%	0.0%	3.3%	85.8%
		(–)	(–)	(–)	(–)	(–)	(–)	(–)	(–)	(–)

**Table 2:** Summary of the accuracy assessment results (ID001 and ID002): overall accuracy OA values (percent) for the sixteen mixture characterizations (SAM, spectral angle mapper; MD, minimum distance; MhlD, Mahalanobis distance; ML Maximum Likelihood.)

*OA*	Pixel-Wise Classification				Particle-Wise Classification			
	SAM	MD	MhlD	ML	SAM	MD	MhlD	ML
*R*	65.7%	49.7%	83.0%	81.6%	91.5%	61.6%	97.2%	96.0%
$R_{SG}$	54.5%	73.0%	87.7%	81.8%	93.1%	96.2%	96.5%	96.6%
$R_{M}$	45.8%	46.0%	68.1%	79.5%	59.1%	59.1%	96.4%	96.6%
$R_{SNV}$	49.0%	49.5%	61.2%	71.3%	90.3%	60.0%	96.3%	95.5%

**Table 3:** Summary of the accuracy assessment results (ID001 and ID002): kappa coefficient KC values for the sixteen mixture characterizations.

*KC*	Pixel-Wise Classification				Particle-Wise Classification			
	SAM	MD	MhlD	ML	SAM	MD	MhlD	ML
*R*	0.55	0.27	0.77	0.75	0.93	0.41	0.96	0.94
$R_{SG}$	0.40	0.64	0.83	0.75	0.90	0.94	0.95	0.95
$R_{M}$	0.30	0.30	0.57	0.72	0.41	0.41	0.95	0.95
$R_{SNV}$	0.33	0.32	0.49	0.62	0.86	0.42	0.95	0.93

**Table 4:** Example of the confusion matrix (ID001 and ID002) for the pixel-wise classification obtained with the $R_{SG}$-MhlD combination (percent).

		Reference				
		CuZn	Fe	Cu	Al	Ni
Classification	CuZn	93.0%	0.5%	0.0%	0.0%	12.5%
	Fe	0.0%	74.0%	0.0%	15.0%	15.5%
	Cu	0.5%	0.0%	98.5%	0.0%	0.0%
	Al	0.5%	10.0%	0.0%	84.5%	1.5%
	Ni	6.0%	15.5%	1.5%	0.5%	70.5%

**Table 5:** Example of confusion matrix (ID001 and ID002) for the particle-wise classification obtained with the $R_{SG}$-MhlD combination (percent).

		Reference				
		CuZn	Fe	Cu	Al	Ni
Classification	CuZn	100.0%	0.5%	0.0%	0.0%	23.9%
	Fe	0.0%	90.7%	0.0%	0.0%	0.0%
	Cu	0.0%	9.27%	100.0%	0.0%	0.0%
	Al	0.0%	0.0%	0.0%	100.0%	0.0%
	Ni	0.0%	0.0%	0.0%	0.0%	76.9%
